# Supplementary material for: The paleoclimatic footprint in the soil carbon stock of the Tibetan permafrost region
Source: Nat Commun. 2019 Sep 13;10:4195. doi: 10.1038/s41467-019-12214-5 (PMC6744502; doi:10.1038/s41467-019-12214-5)
Supplement: Supplementary file 1 — Supplementary Information [file 41467_2019_12214_MOESM1_ESM.pdf]

Supporting Information

# The paleoclimatic footprint in the soil carbon stock of the Tibetan permafrost region

Ding et al.

## Supplementary Figures

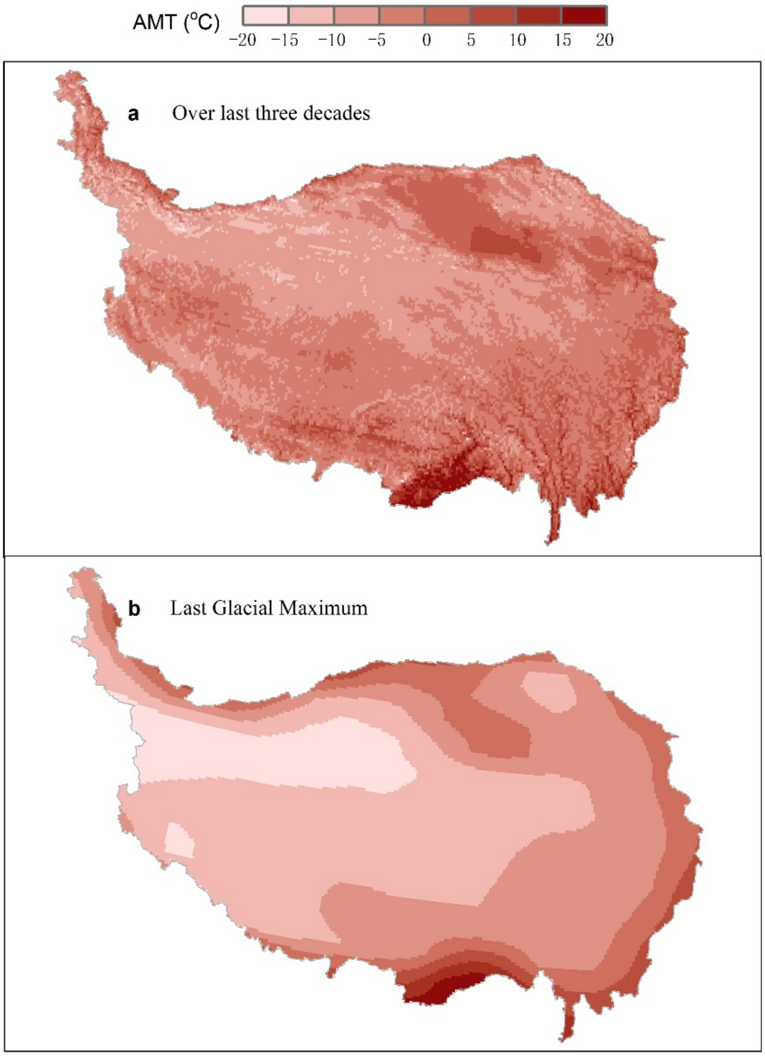


**Supplementary Figure 1.** Spatial patterns of annual mean temperature (AMT) during modern time (**a**) and Last Glacier Maximum (**b**).

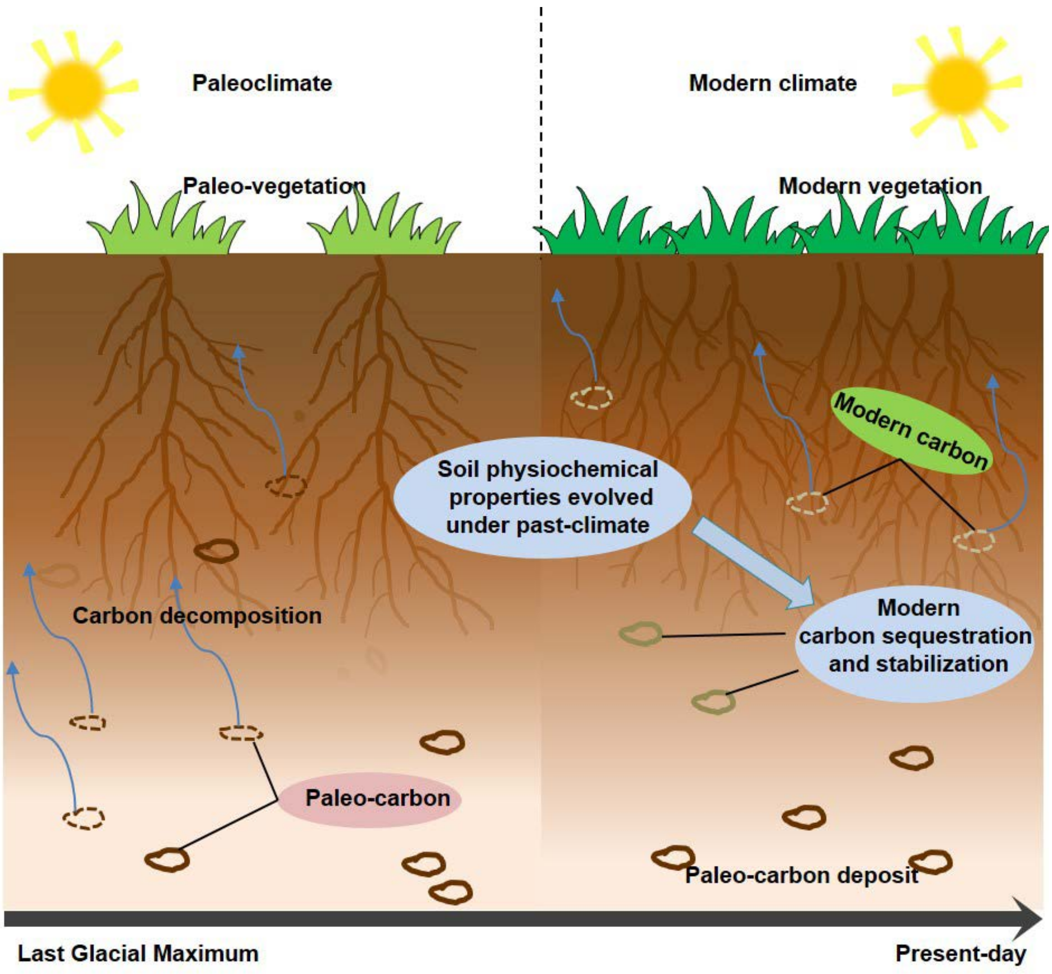


**Supplementary Figure 2.** Schematic of paleoclimate legacy on present-day soil carbon stock through direct paleo-carbon deposit or indirectly changing soil physiochemical properties.

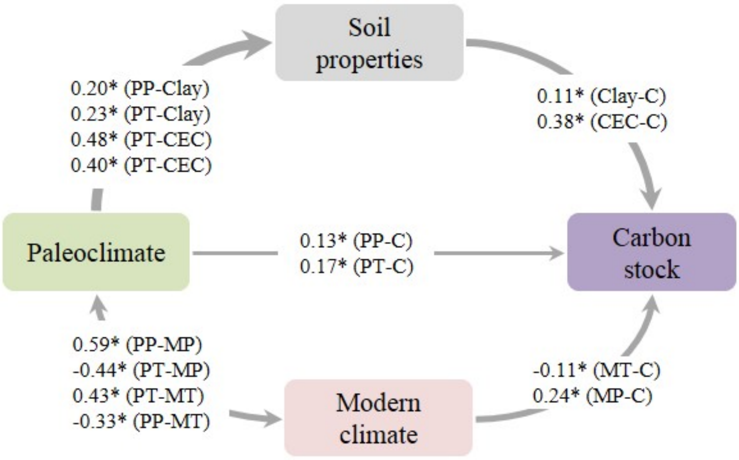


**Supplementary Figure 3.** Structural Equation Modeling (SEM) for the top 30 cm soil carbon stock across the Tibetan permafrost regions. The SEM had whole-model *P* = 0.24, $\chi^2$ = 1.38, RMSEA = 0.02, and AIC = 55.38. The specification of an appropriate SEM model requires that the difference between the SEM simulation and the observations is not statistically significant ($P > 0.05$). Model parameter optimization was further pursued by decreasing values of $\chi^2$, RMSEA, and AIC and to increase the *P* value so as to improve the model fit to the observations[1, 2]. The numbers in the figure are standardized path coefficients, which can reflect the relative importance of the predicted variables within the model. * denotes statistically significant at the 0.05 probability level. The double-headed arrows denote the covariance between related variables. PP means paleo-precipitation conditions over the LGM; PT means paleo-temperature conditions over the LGM; MP means modern precipitation; MT means modern temperature.

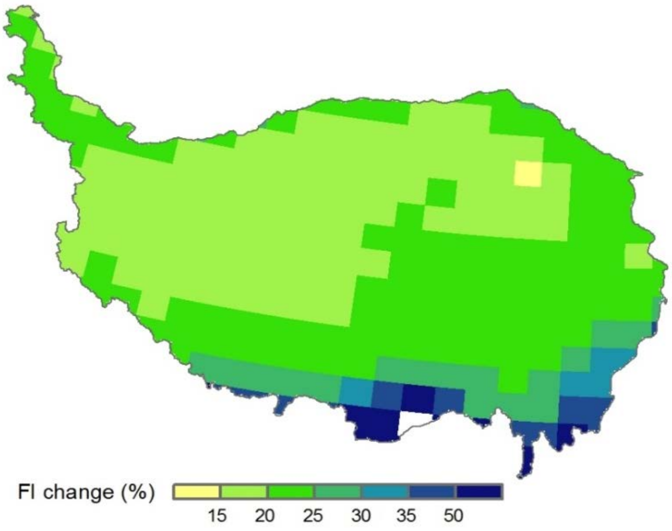


**Supplementary Figure 4.** Spatial pattern of the relative change of freezing index (FI) from Last Glacial Maximum to mid-Holocene.

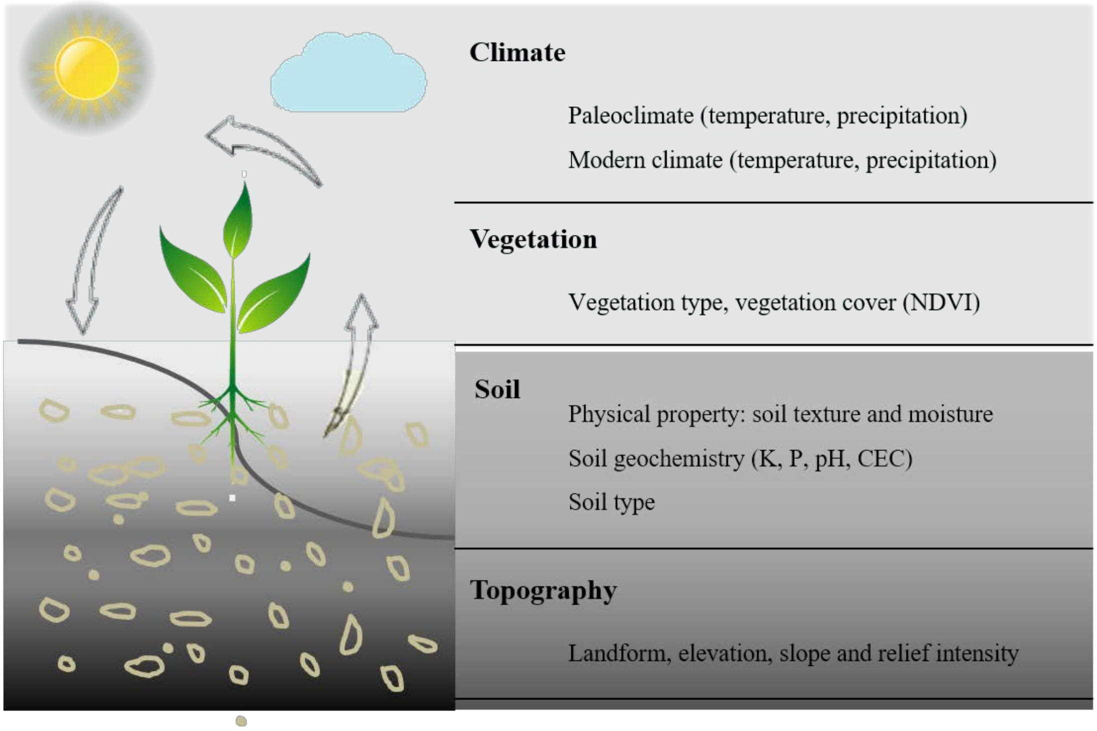


**Supplementary Figure 5.** Diagram of factors controlling soil carbon patterns, which were used for machine learning model algorithms to estimate soil carbon stock over Tibetan permafrost regions.

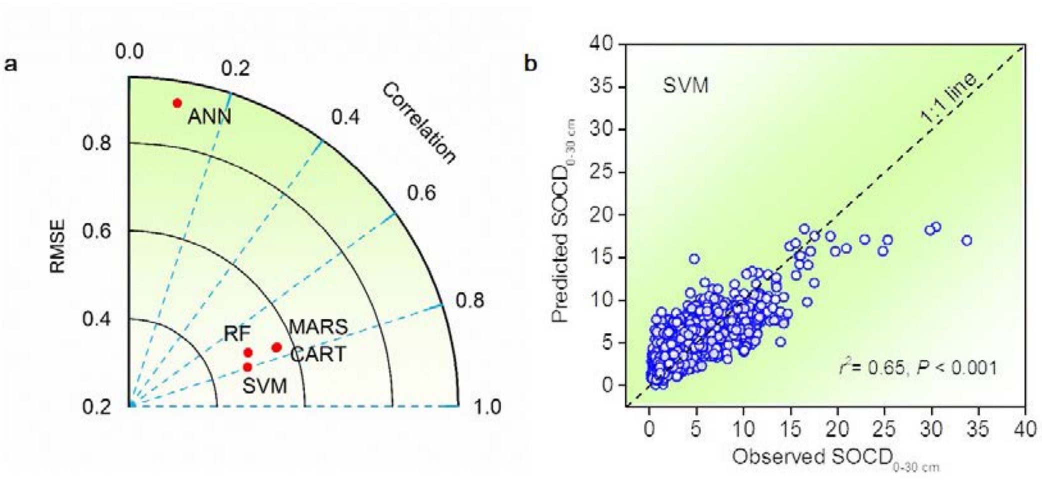


**Supplementary Figure 6. a** Comparisons of prediction performance of multiple machine learning methods for top 30 cm SOCD ($SOCD_{0\text{-}30\ cm}$), based on "Leave-one-out" cross-validation. **b** Scatter plot of the observed and the predicted SOCD for Support Vector Machine (SVM) model only because of its better performance in predicting SOCD than other approaches. Root mean square error (RMSE) was used to measure the differences between predicted and observed SOCD. RF, Random Forest; ANN, Artificial Neural Network; CART, Classification and Regression Trees; MARS, Multivariate Adaptive Regression Splines. The dashed line in **b** is the 1:1 line.

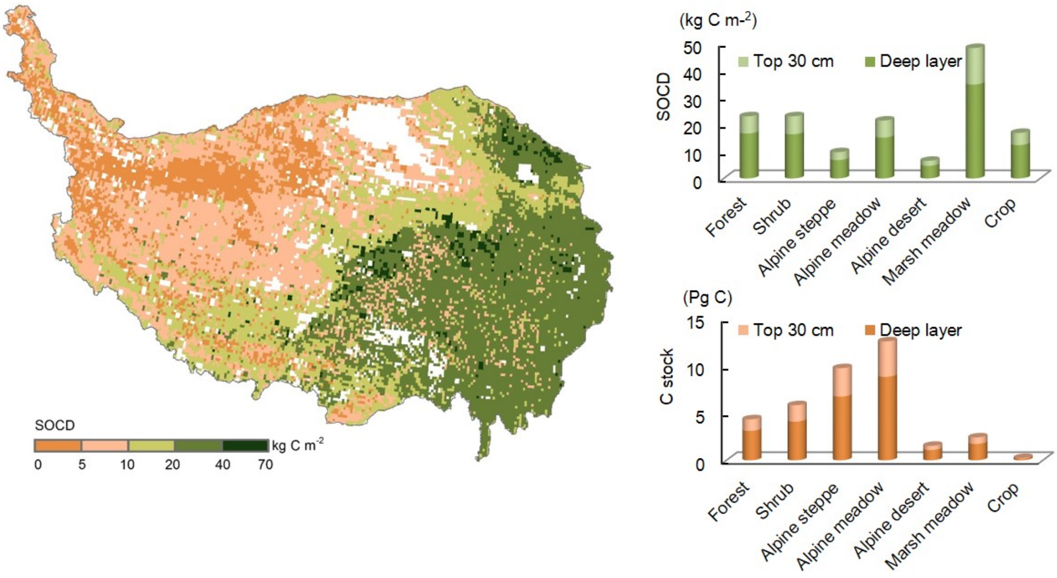


**Supplementary Figure 7.** Soil carbon stock to 3 m depth for permafrost regions of the Tibetan plateau.

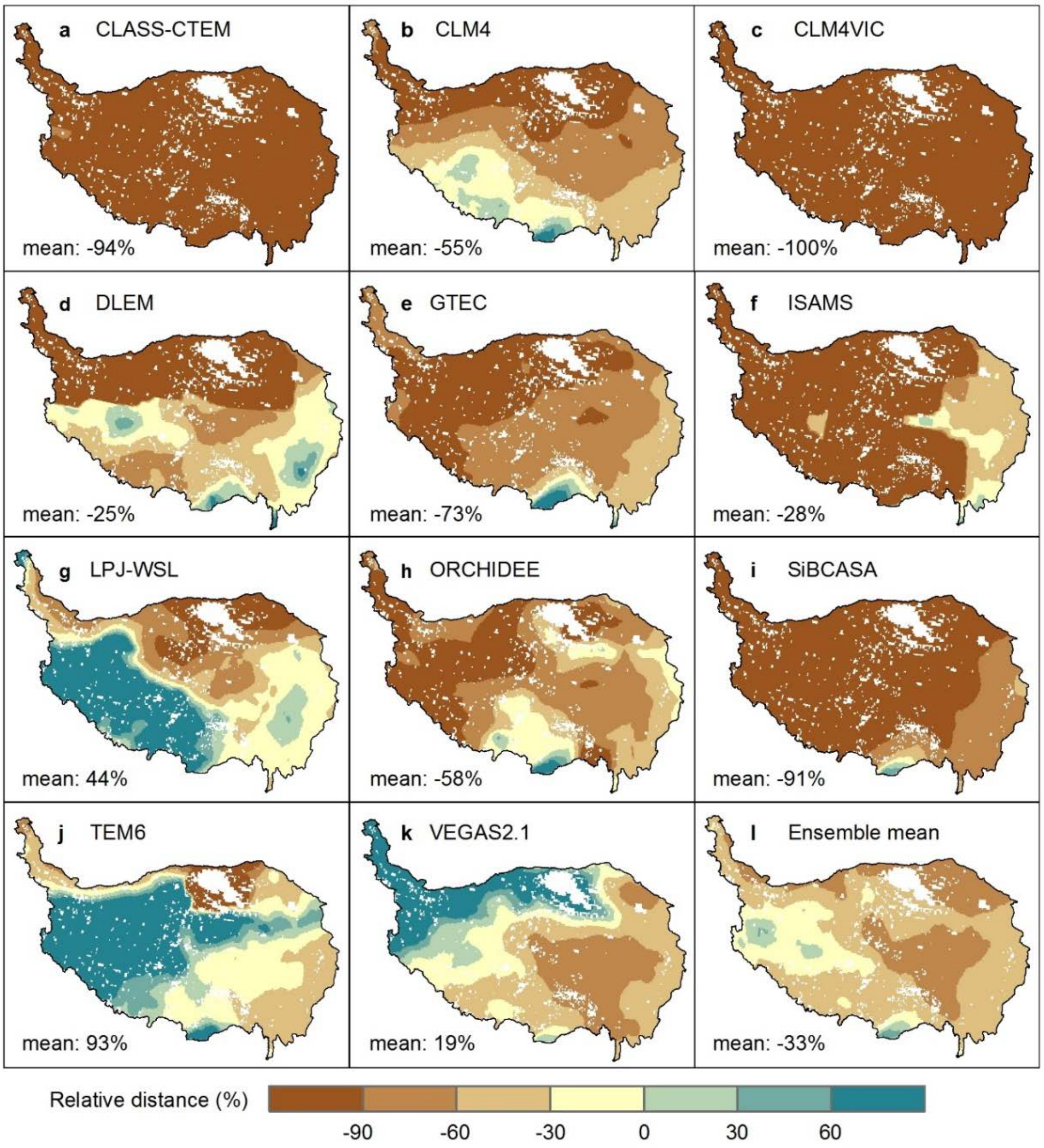


**Supplementary Figure 8.** Spatially explicit multi-scale averaged relative distance between the soil carbon stock simulated by each model (**a-k**) or the ensemble mean (**l**) and our estimate, using the Comparison Map Profile method[3].

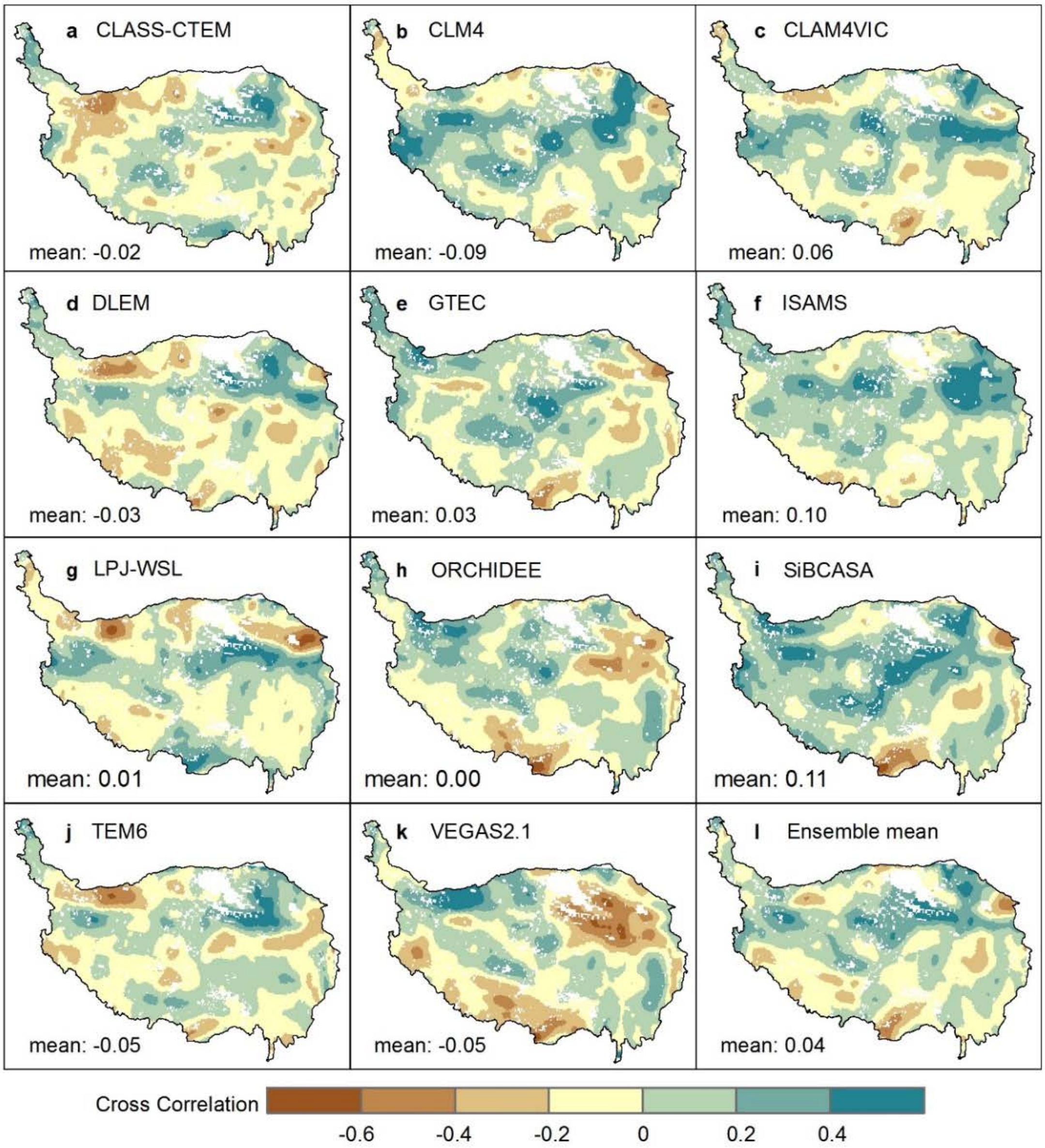


**Supplementary Figure 9.** Spatially explicit multi-scale averaged cross-correlation between the soil carbon stock simulated by each model (**a**-**k**) or the ensemble mean (**l**) and our estimate, using the Comparison Map Profile method[3].

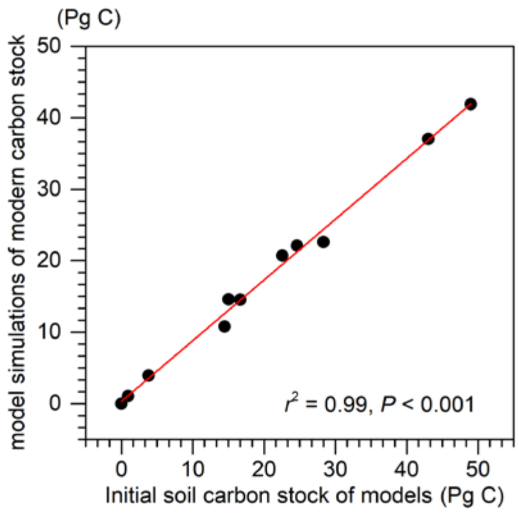


**Supplementary Figure 10.** Dependence of modern soil organic carbon stock simulations on initial soil carbon stocks (mean values during the 1901-1910) across the 11 ecosystem models.

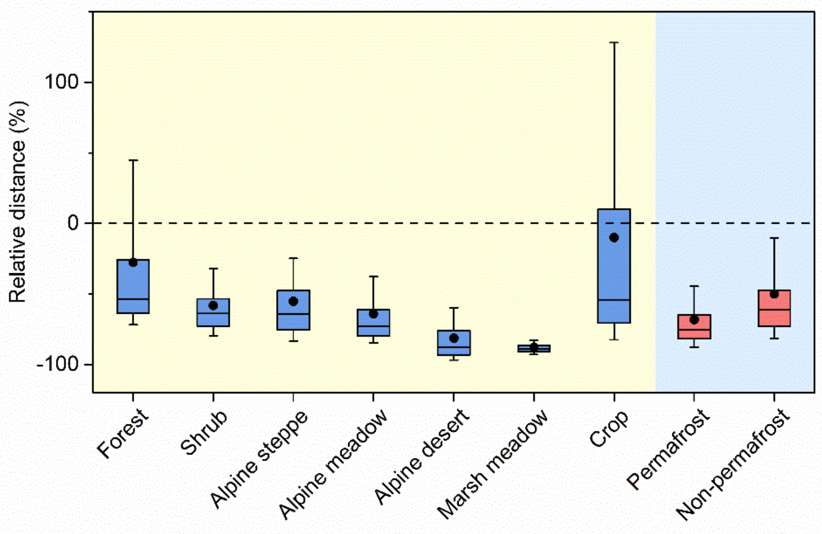


**Supplementary Figure 11.** Bias in soil carbon stock based upon vegetation specific region as indicated by the relative distance of weighted ensemble mean of the 11 model outputs as compared to the new observational estimate in this study. The black dots and lines inside the boxes represent mean and median values, and box ends represent 25$^{th}$ to 75$^{th}$ quartile range, and whistlers denote 10$^{th}$ to 90$^{th}$ quartile range.

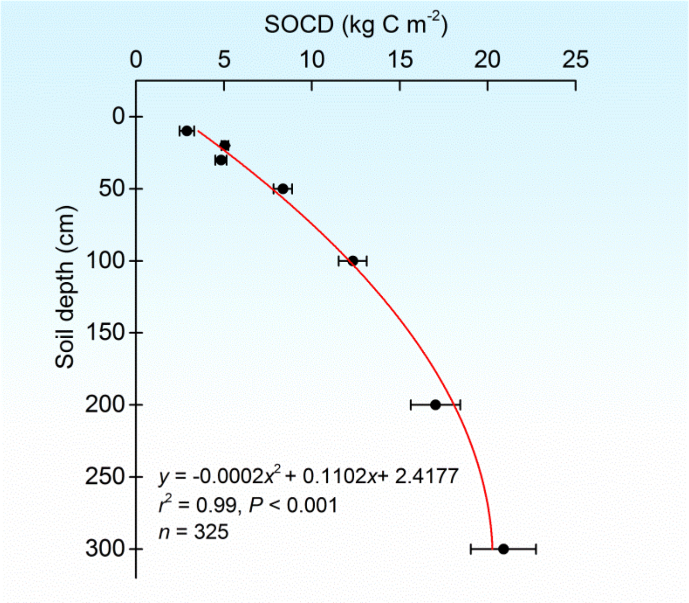


**Supplementary Figure 12.** Relationship of soil organic carbon density (SOCD) with soil depth, based on the 325 deep sampling sites.

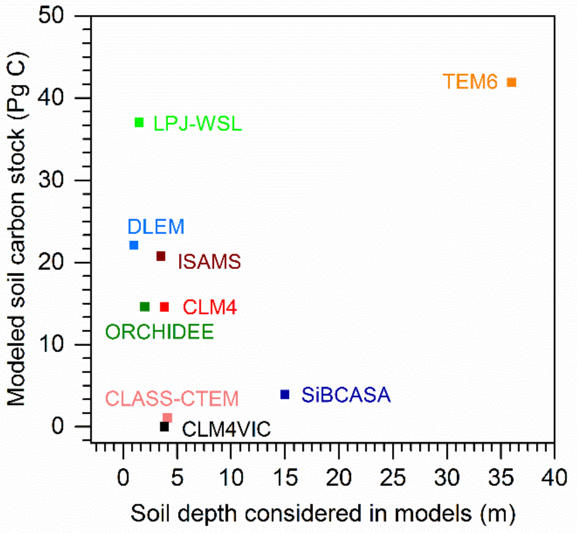


**Supplementary Figure 13.** Scatter plot of the soil carbon stocks and the soil depths considered in the 11 ecosystem models.

## Supplementary Tables

**Supplementary Table 1.** Parameter estimates and significance tests for the structural equation model (SEM) for SOCD for the top 30 cm layer ($n = 1114$)

| | | | SC | UC | SE | *P*-value |
|---|---|---|---|---|---|---|
| Clay content | <--- | T-LGM | 0.234 | 0.246 | 0.035 | *** |
| Clay content | <--- | P-LGM | 0.196 | 0.201 | 0.038 | *** |
| CEC | <--- | T-LGM | 0.481 | 1.090 | 0.071 | *** |
| Clay content | <--- | T-Modern | 0.13 | 0.14 | 0.032 | *** |
| CEC | <--- | P-LGM | 0.402 | 0.888 | 0.074 | *** |
| SOCD | <--- | Clay content | 0.11 | 0.186 | 0.047 | *** |
| SOCD | <--- | T- Modern | -0.109 | -0.200 | 0.05 | *** |
| SOCD | <--- | P- Modern | 0.235 | 0.520 | 0.079 | *** |
| SOCD | <--- | T-LGM | 0.169 | 0.300 | 0.06 | *** |
| SOCD | <--- | P-LGM | 0.133 | 0.230 | 0.068 | *** |
| SOCD | <--- | CEC | 0.381 | 0.299 | 0.024 | *** |

SC represents standardized path coefficient; UC represents unstandardized path coefficient; SE is standard error; *** indicates *P*-value < 0.001. T, temperature; P, precipitation; CEC, Cation exchange capacity; LGM, Last Glacial Maximum.

**Supplementary Table 2.** The soil carbon age data from soil radiocarbon ($^{14}C$) dating studies on the Tibetan Plateau

| Soil depth (m) | Age (yr BP) | Location | Reference |
|---|---|---|---|
| 0.105 | > 1958 AD | 31.32°N, 92.06°E | Kaiser *et al.*, 2008[4] |
| 0.15 | >1958 AD | 31.59°N, 91.48°E | Kaiser *et al.*, 2008[4] |
| 0.1 | >1958 AD | 31.32°N, 92.06°E | Kaiser *et al.*, 2008[4] |
| 0.14 | 723 | 31.86°N, 93.09°E | Kaiser *et al.*, 2008[4] |
| 0.17 | 1882 | 31.76°N, 92.63°E | Kaiser *et al.*, 2008[4] |
| 0.14 | 395 | 31.32°N, 92.06°E | Kaiser *et al.*, 2008[4] |
| 0.29 | 798 | 31.59°N, 91.48°E | Kaiser *et al.*, 2008[4] |
| 0.18 | 321 | Not available | Kaiser *et al.*, 2008[4] |
| 0.25 | 8894 | 37.54°N, 101.33°E | Kaiser *et al*. 2007[5] |
| 0.1 | 3295 | 32.98°N, 98.10 °E | Jin *et al*. 2007[6] |
| 0.2 | 3925 | 34.48°N, 100.23 °E | Jin *et al*. 2007[6] |
| 0.3 | 3270 | 30.07°N, 90.55°E | Jin *et al*. 2007[6] |
| 0.4 | 7207 | 35.88°N, 94.50°E | Jin *et al*. 2007[6] |
| 0.5 | 1080 | 34.91°N, 97.71°E | Jin *et al*. 2007[6] |
| 0.3 | 3270 | 30.08°N, 90.55°E | Jin *et al*. 2007 |

**Supplementary Table 3.** Comparisons of soil depths and simulations of soil organic carbon stock among the 11 ecosystem models. NA indicates not available

| Model | Soil depth (m) | Soil organic carbon stock (Pg C) |
|---|---|---|
| CLASS-CTEM | 4.1 | 1.06 |
| CLM4 | 3.82 | 14.55 |
| CLM4VIC | 3.82 | 0.01 |
| DLEM | 1 | 22.09 |
| GTEC | NA | 10.81 |
| ISAMS | 3.5 | 20.72 |
| LPJ-WSL | 1.5 | 37.03 |
| ORCHIDEE | 2 | 14.59 |
| SiBCASA | 15 | 3.92 |
| TEM6 | 36 | 41.90 |
| VEGAS2.1 | NA | 22.60 |

## Supplementary Discussion

We observed significant variability in the performance of the ecosystem models. For instance, the LPJ-WSL and TEM6 models simulated a soil carbon stock that is statistically close to our estimate, but other models, such as CLASS-CTEM and CLM4VIC, showed a considerable underestimation (Figure 4). This wide range of soil carbon stock simulation by ecosystem models could be related to differences in the models' representation of soil carbon input (as indicated by NPP) and the turnover time of the soil carbon pool (given by the total soil carbon stock divided by soil heterotrophic respiration). For instance, the mean NPP (0.28 Pg C $yr^{-1}$) simulated by models with the lower soil carbon stock such as CLASS-CTEM and CLM4VIC, is much lower than the mean simulated NPP (0.44 Pg C $yr^{-1}$) from models with higher soil carbon stock such as LPJ-WSL and TEM6. Moreover, the mean modelled turnover time derived from CLASS-CTEM and CLM4VIC (12 years, mean for the two models) is much less than that derived from LPJ-wsl and TEM6 (234 years, mean for the two models). In addition, the soil layer depth tends to vary across models (Supplementary Table 3), but it appears that the soil depth is not a major source of the model spread in the simulation of soil carbon stock, since there was no significant correlation between the simulated soil carbon stock and soil depth across 11 models ($r^2$ = 0.07, $P$ = 0.25; Supplementary Figure 13).

**Supplementary References**


1. Grace, J. B. *Structural Equation Modeling and Natural Systems* (Cambridge University Press, 2006).

2. Shipley, B. *Cause and Correlation in Biology* (Cambridge University Press, 2000).

3. Gaucherel, C., Alleaume, S. & Hely, C. The Comparison Map Profile Method: A Strategy for Multiscale Comparison of Quantitative and Qualitative Images. *IEEE T. Geosci. Remote* **46**, 2708-2719 (2008).

4. Kaiser, K., *et al.* Turf-bearing topsoils on the central Tibetan Plateau, China: Pedology, botany, geochronology. *Catena* **73**, 300-311 (2008).

5. Kaiser, K., Schoch, W. H. & Miehe, G. Holocene paleosols and colluvial sediments in Northeast Tibet (Qinghai Province, China): Properties, dating and paleoenvironmental implications. *Catena* **69**, 91-102 (2007).

6. Jin, H. J., Chang, X. L. & Wang, S. L. Evolution of permafrost on the Qinghai-Xizang (Tibet) Plateau since the end of the late Pleistocene. *J. Geophys. Res.* **112**, F02S09 (2007).
